# Supplementary material for: Learning curve and surgical outcome of robotic assisted colorectal surgery with ERAS program
Source: Sci Rep. 2022 Nov 29;12:20566. doi: 10.1038/s41598-022-24665-w (PMC9709162; doi:10.1038/s41598-022-24665-w)
Supplement: Supplementary file 1 — Supplementary Information 1. [file 41598_2022_24665_MOESM1_ESM.zip › LSC Raw data-Ñ[▒K/074/P_ñw╝╨░O▒Kñσ.pdf]

[redacted]

Pathologic diagnosis:
1. Rectum, laparoscopic total mesorectal resection --- Adenocarcinoma, moderately differentiated. Tumor invaded to perirectal soft tissue and metastasized to perirectal lymph nodes (6/16) with multiple tumor deposits. The proximal cut end is free of tumor.
2. Cut end, distal, resection --- Mild hemorrhoid and unremarkable squamous mucosa.
3. IMA root, dissection --- Unremarkable lymph node (0/1) and fibrovasculoadipose tissue.

Ancillary study for diagnosis:
IHC stains for PMS2 and MSH6 are done on section B. Both markers are preserved.

Prognostic and predictive factor:
1. Tumor size: 4.5x3.5 cm.
2. Depth of invasion: Perirectal soft tissue
3. Histologic grade: G2: Moderately differentiated.
4. Angiolymphatic invasion: Present.
5. Perineural invasion: Present.
6. Tumor deposits (discontinuous extramural extension): Present, specify number of deposits: 7.
7. Macroscopic tumor perforation: Not identified.
8. Surgical Margins: a) Proximal Margin: Uninvolved by invasive carcinoma, distance of tumor from margin: 17.9 cm. b) Distal Margin: Uninvolved by invasive carcinoma, distance of tumor from margin: Cannot be assessed due to seperated specimen. c) Radial or Mesenteric Margin: Involved by invasive carcinoma (tumor present 0-1 mm from margin).
9. Lymph node status: Involved (6/17).
10. Extranodal involvement: Present.
11. Tumor regression grading S/P CCRT: No known presurgical therapy.
12. Type of polyp in which invasive carcinoma arose: Not identified.
13. Additional pathologic findings: Not identified.
14. Pathological TNM Stage: pT3N2a (According to the eighth edition, American Joint Committee on Cancer Staging Guidelines for Tumors).
15. TNM descriptors: Not applicable.

Gross description:
The specimen consists of 1) a segment of colon, 21.4 cm in length and 4.5 cm in proximal cut end circumference, 4.2 cm in distal circumference, and perirectal soft tissue. A gray tan firm annular elevated and centrally ulcerative tumor, 4.5x3.5 cm, is noted, very close to the distal cut end. On cross section, the tumor invades to the mesocolic soft tissue with 2 mm to the radial margin. The proximal cut end is free of tumor grossly. 2) distal cut end, a piece of tan mucosa-covered soft tissue, 2.1x1.8x0.8 cm. 3) IMA root, a piece of tan yellow soft tissue, 3.8x2.1x0.7 cm. Representative part for section: A) proximal cut end B-E) tumor and deep margin F) specimen 2 G) specimen 3 H-K) lymph nodes.

Note:
1. The interpretation of MMR panel and recommended studies are as below: No loss of nuclear expression of MMR proteins: low probability of microsatellite instability-high (MSI-H). Please note: There are exceptions to the above IHC interpretations. These results should not be considered in isolation, and clinical correlation with genetic counseling is recommended to assess the need for germline testing.
2. This case has been peer reviewed by two doctors.
#T-59600_2 #M-81403_2 2 1105 S0000F
#T-C4560_2 #M-81406_2 2 1105 000000

已電子簽章

敬告：本單僅供醫師參考使用，不具任何申請效力！！
請病患妥為保管，若不慎遺失需自負個人資料外洩責任！
